# Supplementary material for: An In Situ Study on Nanozyme Performance to Optimize Nanozyme-Strip for Aβ Detection
Source: Sensors (Basel). 2023 Mar 24;23(7):3414. doi: 10.3390/s23073414 (PMC10098967; doi:10.3390/s23073414)
Supplement: Supplementary file 1 [file sensors-23-03414-s001.zip › sensors-2257857-supplementary.pdf]

Supporting information

An In Situ Study on Nanozyme Performance to Optimize Nanozyme-Strip for Aβ Detection

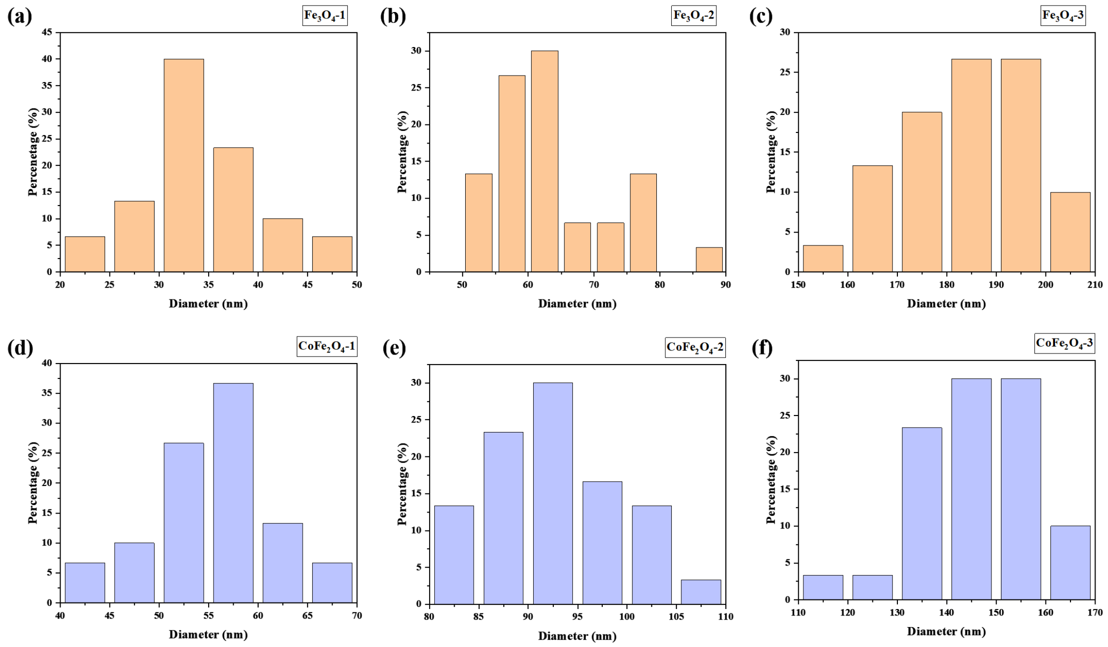

Figure S1. Particle size distribution of nanozymes (a) Fe_3O_4-1 nanozyme (b) Fe_3O_4-2 nanozyme (c) Fe_3O_4-3 nanozyme (d) $CoFe_2O_4$-1 nanozyme (e) $CoFe_2O_4$-2 nanozyme (f) $CoFe_2O_4$-3 nanozyme.

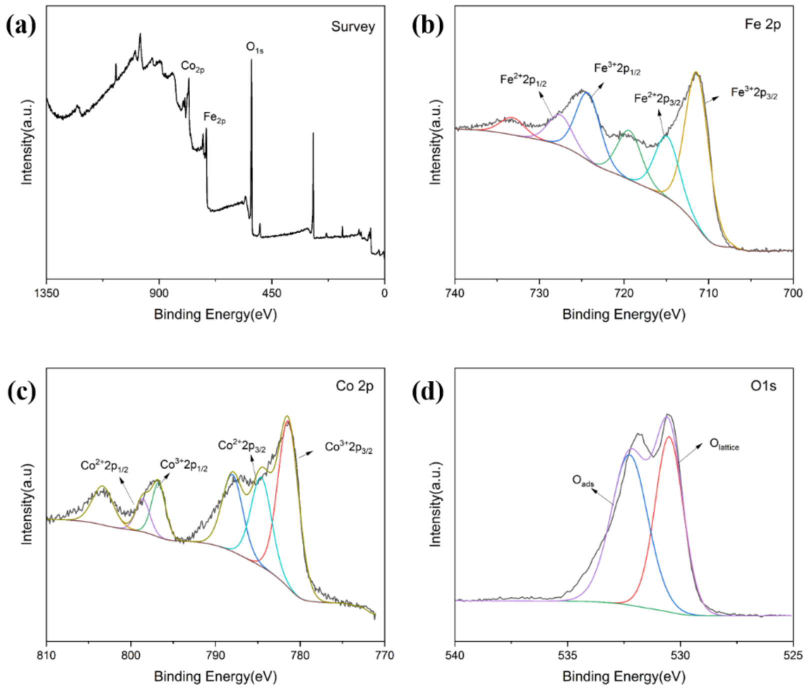

Figure S2. Characterization of $CoFe_2O_4$ nanozyme by XPS. (a) Survey spectra of $CoFe_2O_4$. (b) High-resolution spectra of Fe 2p. (c) High-resolution spectra of Co 2p. (d) High-resolution spectra of O 1s.

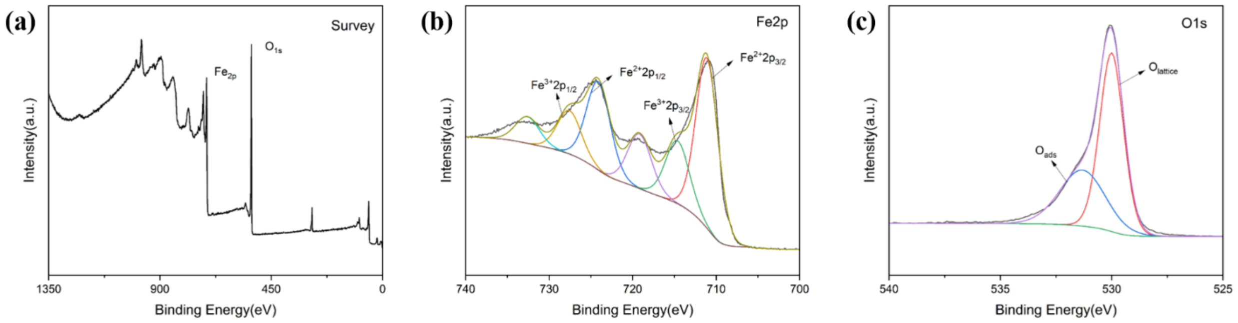

Figure S3. Characterization of Fe_3O_4 nanozyme by XPS. (a) Survey spectra of Fe_3O_4. (b) High-resolution spectra of Fe 2p. (c) High-resolution spectra of O 1s.

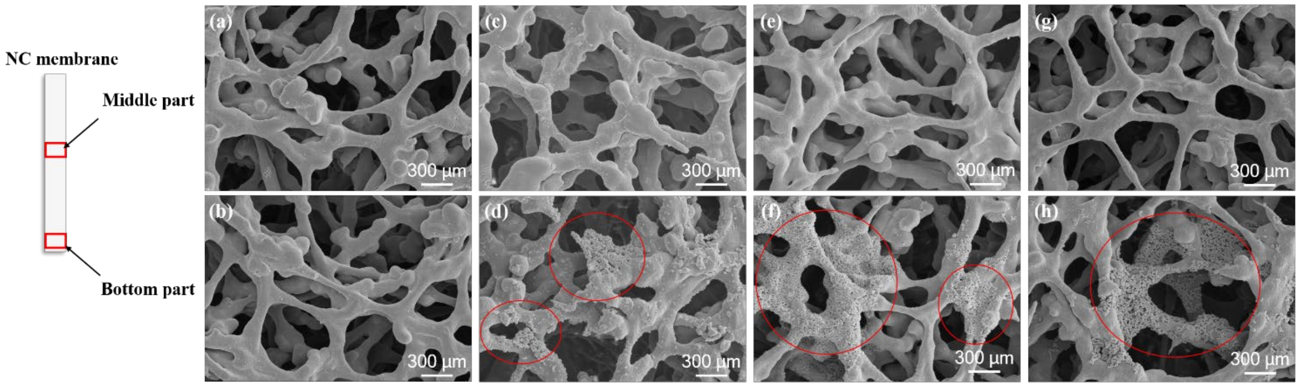

Figure S4. Characterization of Fe_3O_4 nanozymes on NC membrane by SEM. (**a**) Middle part of blank NC membrane. (**b**) Bottom part of blank NC membrane. (**c**) Middle part of Fe_3O_4-1-NC membrane. (**d**) Bottom part of $CoFe_2O_4$-1-NC membrane. (**e**) Middle part of Fe_3O_4-2-NC membrane. (**f**) Bottom part of Fe_3O_4-2-NC membrane. (**g**) Middle part of Fe_3O_4-3-NC membrane. (**h**) Bottom part of Fe_3O_4-3-NC membrane.

Table S1. PDI of nanozymes detected by DLS.

	**$CoFe_2O_4$-1**	**$CoFe_2O_4$-2**	**$CoFe_2O_4$-3**	**Fe_3O_4-1**	**Fe_3O_4-2**	**Fe_3O_4-3**
**PDI**	0.053	0.052	0.066	0.266	0.257	0.274
